# Assessment of Quantitative Genetic Distances Supports the Separation of H17N10 and H18N11 Subtypes of Influenza A Virus into a Distinct Species

**DOI:** 10.3390/v18080838

**Published:** 2026-07-30

**Authors:** Ilya A. Volkhin, Mariia A. Dashian, Alexander N. Lukashev, German A. Shipulin, Andrei A. Deviatkin

**Affiliations:** 1 Faculty of Biology, Lomonosov Moscow State University, 119234 Moscow, Russia; 2 Federal State Budgetary Institution ‘Centre for Strategic Planning and Management of Biomedical Health Risks’ of the Federal Medical and Biological Agency, 119121 Moscow, Russia; 3 Department of Biomedicine, Pirogov Medical University, 117997 Moscow, Russia; 4 Martsinovsky Institute of Medical Parasitology, Tropical and Vector Borne Diseases, Sechenov First Moscow State Medical University, 119435 Moscow, Russia; 5 Laboratory of Postgenomic Technologies, Izmerov Research Institute of Occupational Health, 105275 Moscow, Russia

**Keywords:** *Alphainfluenzavirus*, bat influenza viruses, influenza A virus, H17N10, H18N11, species demarcation, genetic distance, dN/dS, taxonomy

## Abstract

The taxonomic status of the H17N10 and H18N11 influenza A viruses isolated from bats remains unclear due to the absence of quantitative classification criteria at this taxonomic level. A total of 3328 representative IAV genomes, encompassing all eight protein-coding segments, were analysed. Various genetic distance-based metrics were assessed at the pairwise level, including intra- and intergroup nucleotide distances, dN/dS ratios, and transition/transversion ratios, to facilitate the differentiation of the *Alphainfluenzavirus* genus into distinct taxa. Pairwise distances for seven of the eight segments (PB2, PB1, PA, NP, M, NA, NS) consistently differentiated the H17–H18 group from H1–H16. Across segments, intergroup nucleotide divergence was consistently above a lower bound of ~25%, with segment-specific values extending to higher levels (up to ~40% in PB2 and PA), while intragroup divergence remained substantially lower. The HA segment did not conform to this pattern, which is consistent with the hypothesis of ancient reassortment. The distribution of pairwise dN/dS values for the PB2, PB1, PA, and NP segments is evidently bimodal. Intergroup comparisons were consistently higher across all segments, whereas intragroup values remained lower. A similar lower boundary of approximately 0.12 was observed across segments, while the upper range of intergroup values varied by gene. Overall, the results support a consistent gene-specific separation pattern. Previously demonstrated absence of reassortment compatibility between bat viruses (H17–H18) and canonical influenza A (H1–H16) viruses indicates that these lineages have evolved independently over an extended period. These consistent genomic patterns provide support for the hypothesis that H17N10 and H18N11 viruses may represent a separate species within the genus *Alphainfluenzavirus*.

## 1. Introduction

The Orthomyxoviridae family comprises nine genera, which in turn include 21 virus species [1]. Four genera, *Alphainfluenzavirus*, *Betainfluenzavirus*, *Gammainfluenzavirus* and *Deltainfluenzavirus*, are represented by influenza viruses [1]. Notably, all these genera contain a sole species: *Alphainfluenzavirus influenzae* (Influenza A virus, IAV), *Betainfluenzavirus influenzae* (Influenza B virus, IBV), *Gammainfluenzavirus influenzae* (Influenza C virus, ICV), and *Deltainfluenzavirus influenzae* (Influenza D virus, IDV), respectively [2]. All these viruses can infect human respiratory epithelial cells under experimental conditions [3,4]. Influenza A and B viruses are responsible for seasonal epidemics in humans; however, only influenza A viruses have been associated with pandemic spread. As a result, IAV and IBV are represented by substantially more genomic sequences than ICV and IDV, indicating their higher epidemiological significance and surveillance intensity.

The genomes of IAV and IBV are segmented and consist of eight RNA segments [5] encoding the subunits of viral RNA-dependent RNA polymerase (PB2, PB1, and PA), internal structural proteins (M1 and NP), surface glycoproteins hemagglutinin (HA) and neuraminidase (NA), and nonstructural proteins, including NS1. The segmented organization of the genome underlies frequent reassortment and determines distinct evolutionary dynamics of influenza virus genome segments.

Historically, influenza virus species recognized by the International Committee on Taxonomy of Viruses (ICTV) were defined as clusters of viruses that replicate as continuous evolutionary lineages and are capable of genetic reassortment with one another [6]. Because genome segment reassortment requires genetic compatibility, reassortment has long been regarded as an important biological indicator of lineage cohesion within influenza viruses. Although the current ICTV framework employs a broader species concept based on multiple lines of evidence, reassortment compatibility remains an important biological characteristic when evaluating evolutionary relationships among segmented viruses.

Virus proteins encoded by different genome segments are subject to distinct evolutionary pressures. The surface glycoproteins HA and NA are under intense immune-mediated selection [7,8], whereas internal proteins involved in genome replication and viral RNA transport are generally more evolutionarily constrained. One exception is the nonstructural protein NS1 that counteracts host innate immune responses by inhibiting type I interferon signaling [9] and is frequently the subject of adaptive evolution.

The classification of IAV into HA and NA subtypes was originally based on the antigenic properties of the hemagglutinin and neuraminidase glycoproteins [10]. However, contemporary subtype classification also considers genetic divergence and phylogenetic relationships, particularly when newly identified influenza viruses are characterized. To date, 19 HA subtypes (H1–H19) and 11 NA subtypes (N1–N11) have been identified [11]. The primary natural reservoirs of the H1–H16 and H19 subtypes are waterfowl. These 17 HA subtypes are divided into two groups based on their genetic relatedness: group 1 (H1, H2, H5, H6, H8, H9, H11, H12, H13, H16 and H19) and group 2 (H3, H4, H7, H10, H14, and H15) [12]. It should be noted that the only known H19 isolate, A/Common Pochard/Kazakhstan/KZ52/2008, currently has sequence data available only for HA, and the sequences of its remaining genome segments are unknown [11].

In the 2010s, two new subtypes, H17N10 and H18N11, were isolated from bats [13,14]. These viruses were more divergent from other IAV subtypes circulating in mammals and birds than any previously known IAVs. As a result, the taxonomic status of H17N10 and H18N11 remains controversial. They may be designated as “influenza A-like” to emphasise their difference from previously described strains [2,15], or classified as additional IAV subtypes [16,17]. The absence of rigorous quantitative criteria for the identification of types and subtypes of influenza viruses contributes to inconsistent subtype designation. ICTV defines virus species based on multiple criteria, including genetic, phylogenetic, ecological, and biological characteristics. Therefore, measures of genomic divergence can provide additional quantitative support for identifying independently evolving viral lineages. Notably, not all segments of H17N10 and H18N11 are similarly divergent from other IAV subtypes [14,18]. Indeed, the HA segment of H17N10 and H18N11 viruses is closely related to HA subtypes H1, H2, H5, and H6, while other subtypes formed a separate outgroup. This pattern is compatible with an ancient reassortment event, although alternative explanations (e.g., heterogeneous evolutionary rates) cannot be excluded. This event would have hypothetically produced a reassortant virus with an HA gene from an avian or non-bat mammalian IAV ancestor and the remaining seven segments from a bat lineage IAV ancestor. Therefore, the classification of IAVs based solely on antigenic properties may not fully reflect their evolutionary history. A considerable number of reassortment events have occurred, both between and within subtypes. This has resulted in heterogeneous phylogenetic signals that complicate taxonomic interpretation [19,20,21].

IBVs are classified into Yamagata-like and Victoria-like lineages [22], which are more closely related to each other than two distinct IAV subtypes [23]. Notably, IBVs have no known established animal reservoir. Nevertheless, sporadic detections of IBV have been reported in domestic animals (including horses, pigs, goats, and sheep), companion animals (dogs and guinea pigs), and non-human primates (orangutans, gorillas, and chimpanzees) [24].

Despite the importance of taxonomy for surveillance and evolutionary studies, there is currently no quantitative criteria for defining species, subtypes, or lineages of influenza viruses. One way to classify viruses is by using genetic distances. Such criteria have been proposed for some viruses [25,26,27,28,29]. However, this approach has its limitations [30]. The applicability of such approaches to influenza viruses remains uncertain, given their segmented genome organization, extensive reassortment, and pronounced segment-specific heterogeneity in evolutionary constraints. In addition to nucleotide divergence, the pairwise ratio of nonsynonymous to synonymous substitutions (dN/dS) provides complementary information on the relative contribution of amino acid-changing and silent substitutions during viral evolution. Comparative patterns of pairwise dN/dS values may provide additional evidence for long-term evolutionary separation between viral groups. Transition/transversion ratios represent another measure of nucleotide substitution patterns and can provide additional information on the evolutionary processes shaping viral genomes.

This study aimed to evaluate whether quantitative genomic characteristics can provide additional support for species-level classification within the genus *Alphainfluenzavirus*. We systematically analyzed genome-wide nucleotide divergence, amino acid divergence, pairwise dN/dS ratios, and transition/transversion ratios across all eight coding segments of influenza A and B viruses to assess whether these metrics reveal consistent patterns of evolutionary separation between H17–H18 and H1–H16 influenza A viruses.

## 2. Materials and Methods

### 2.1. Dataset Collection, Filtering and Clustering

A dataset containing 44,102 IAV and 12,816 IBV complete protein-coding sequences (with all eight segments present) was downloaded from the GenBank database in September 2024 and February 2025, respectively. All segments were concatenated in a fixed order (PB2–PB1–PA–HA–NP–NA–M–NS) using an in-house script (available at https://github.com/MarselBrukman/Influenza_alignments/blob/main/Concatenation.py, accessed on 14 July 2026). To reduce redundancy caused by the overrepresentation of closely related influenza A virus isolates, sequences were clustered using CD-HIT-EST version 4.8.1, a greedy clustering algorithm that groups highly similar nucleotide sequences, with a 97% nucleotide identity threshold, and one representative sequence from each cluster was retained [31,32]. This threshold was selected as a compromise between computational efficiency and preservation of the major genomic diversity represented in the dataset. The clustered dataset retained representatives of all HA and NA subtypes included in the original dataset. Importantly, the 3% nucleotide divergence threshold used for clustering is substantially lower than the divergence levels observed between H17–H18 and H1–H16 viruses and therefore does not affect the identification of deep evolutionary separation between these groups. Sequences containing degenerate nucleotides or not annotated to any IAV subtype were excluded.

### 2.2. Nucleotide Sequence Alignment

All segments were aligned separately using the MAFFT v7.525 [33] algorithm. The full-length IAV alignment included 3328 sequences representing all known HA and NA subtypes. The IBV alignment included 18 representative sequences capturing the diversity of the Victoria and Yamagata lineages (alignments available at https://github.com/MarselBrukman/Influenza_alignments, accessed on 14 July 2026). The limited size of the IBV dataset should be considered when interpreting comparisons.

### 2.3. Protein Sequence Generation and Alignment

The protein sequences were generated by translating the corresponding coding regions. For each segment, the primary annotated coding sequence corresponding to the main viral protein was used for translation. Protein alignments for each segment were created using the MAFFT v7.525 algorithm [33].

### 2.4. Pairwise Distance Analyses

The pairwise genetic distance was calculated using the Jukes–Cantor model for nucleotide sequences. Amino acid pairwise distances were estimated by maximum likelihood (dist.ml function with the BLOSUM62 model [34]) and are expressed as the expected number of amino acid substitutions per site, explicitly accounting for multiple substitutions. For example, a distance of 1.5 substitutions per site (150%) indicates an average of 1.5 replacements per alignment position, i.e., pronounced saturation. This measure reflects evolutionary divergence rather than raw sequence similarity.

Heat maps of pairwise nucleotide and amino acid distances were generated and visualized in the R environment. The package ape v.5.8.1 was used for handling and computing phylogenetic and distance matrices, seqinr v.4.2.44 for sequence data processing and translation, scales for color normalization, gdata v.3.0.1 for data manipulation, and ggplot2 v.4.0.3 for visualization of distance distributions and heat map rendering.

### 2.5. Pairwise dN/dS Analysis

For pairwise dN/dS analysis, codon-based alignments of IAV and IBV sequences were generated using the PAL2NAL web application [35]. Pairwise dN/dS ratios were calculated using the computeNeiGojobori.py script (available at https://github.com/AndreiDeviatkin/repo/blob/main/Selection_pressure_profile_suggests_species_criteria_among_tick-borne_flaviviruses/scripts/computeNeiGojobori.py, accessed on 14 July 2026).

To reduce technical noise, only sequence pairs with more than 5% synonymous substitutions relative to the length of the given segment were included, as pairs below this threshold yielded unstable estimates due to a very low synonymous divergence resulting in an enormously high dN/dS ratio. In addition, the strain A/chicken/Shanghai/C2/2012 was excluded from the pairwise dN/dS ratio analysis because it produced highly inconsistent pairwise dN/dS ratio estimates relative to the rest of the dataset. Manual inspection did not reveal obvious quality problems, frameshift errors, or annotation inconsistencies in this sequence. Therefore, A/chicken/Shanghai/C2/2012 was excluded from the dataset as a conservative filtering step to avoid disproportionate influence of a single divergent sequence on the observed distributions.

### 2.6. Transition/Transversion Analysis

An in-house script (available at https://github.com/MarselBrukman/Influenza_alignments/blob/main/transition_transversion_mine.R, accessed on 14 July 2026) was used to calculate transition and transversion counts for each pairwise sequence comparison. For each sequence pair, the numbers of transitions and transversions were obtained, and the transition/transversion ratio (Ti/Tv) was calculated. The proportions of transitions and transversions among all observed substitutions were also calculated. These values were compared with raw nucleotide p-distances calculated using pairwise deletion, and the resulting relationships were visualized separately for intra- and intergroup comparisons.

### 2.7. Maximum-Likelihood Phylogenetic Analysis

To reduce the computational burden of phylogenetic analyses while maintaining representation of the major influenza A virus diversity, CD-HIT-EST software was applied to the sequences from the full-length IAV alignment (*n* = 3328) with a similarity threshold of 90%. The result of this process was the selection of 141 sequences covering all HA and NA subtypes. Maximum-likelihood phylogenetic trees were constructed using iqtree2 version 2.0.7 [36] software. TIM2+F+R10 molecular evolution model with 1000 iterations of ultrafast bootstrap analysis was used.

## 3. Results

To define objective and reproducible taxonomic criteria for influenza viruses, we compared pairwise genetic distances and selection pressures among 3328 representative IAV sequences and 18 representative IBV sequences, each covering all eight protein-coding segments. Notably, although IAV and IBV represent taxonomically equivalent species, their overall genetic diversity differs substantially, resulting in a smaller number of IBV sequences remaining after applying identical filtering thresholds. A limitation of the present study is the substantially smaller size of the influenza B virus dataset compared to influenza A virus. Although the IBV dataset includes representative sequences covering both Victoria-like and Yamagata-like lineages, its reduced sampling density limits the resolution of within-lineage diversity estimates. Therefore, quantitative comparisons of absolute genetic diversity between IAV and IBV should be interpreted with caution.

**Figure 1:**
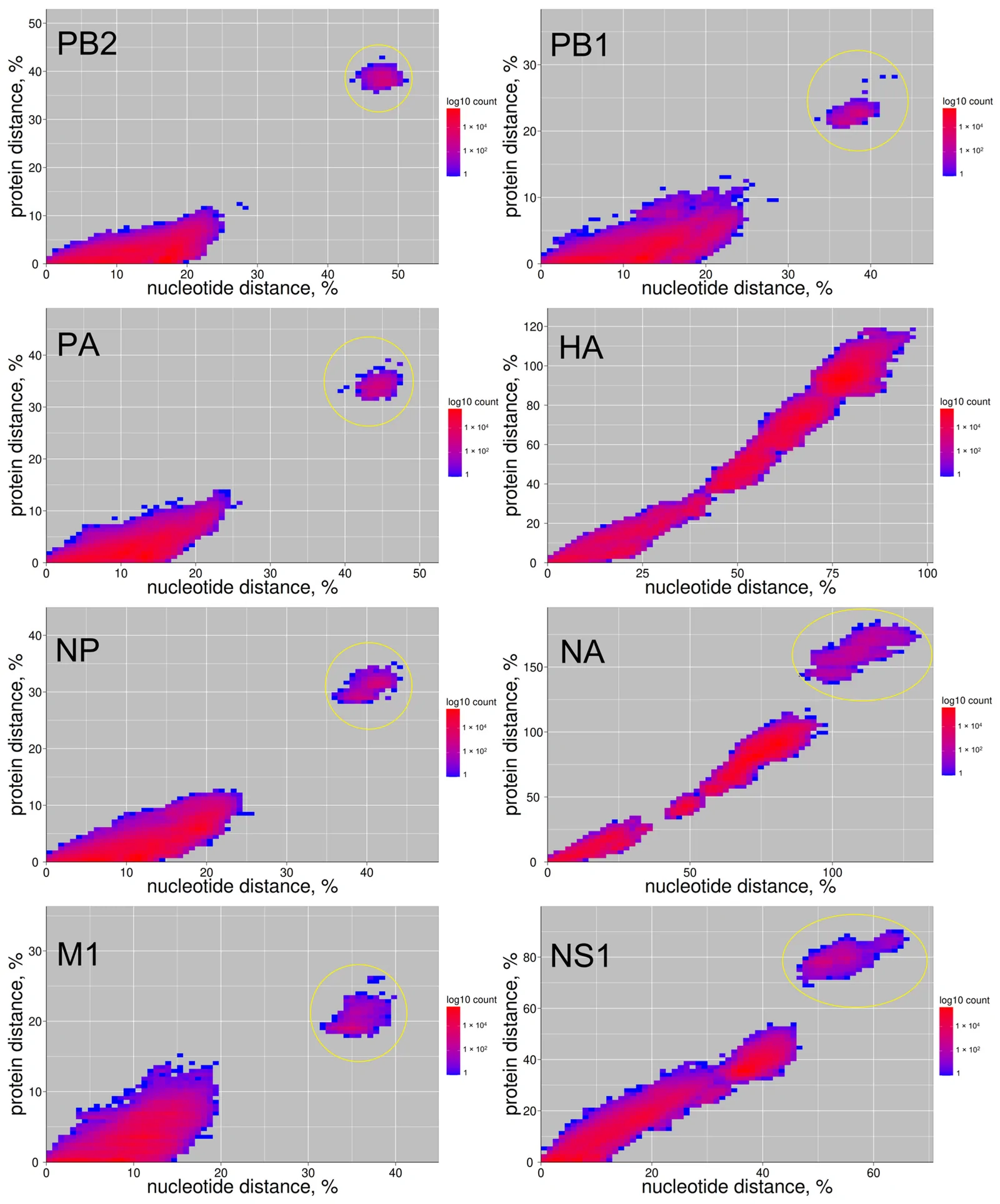
Heat map of pairwise nucleotide and amino acid distances for influenza A virus open reading frame sequences (*n* = 3328). Nucleotide distances were estimated using the Jukes–Cantor model. Amino acid distances were estimated as average substitutions per position estimated using BLOSUM62. Axes show uncorrected amino acid and corrected nucleotide sequence distances; dots correspond to the distances between every two possible sequences in the data set. Color indicates dot density according to the scale. Yellow circles mark the pairs of sequences of H1–H16 versus H17–H18 subtypes. It should be noted that the axis scales differ across viral gene segments due to segment-specific levels of evolutionary divergence.

**Figure 2:**
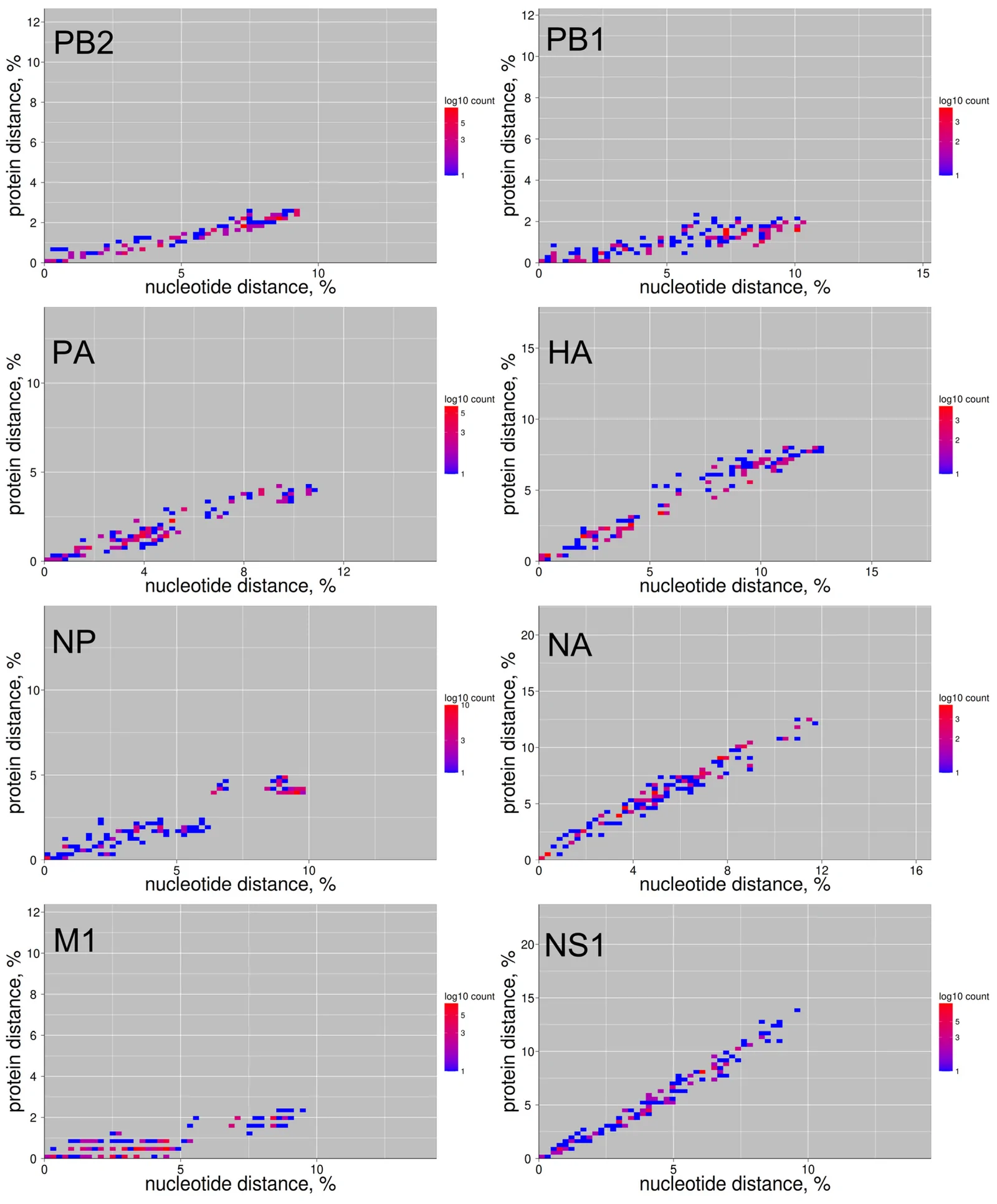
Heat map of pairwise nucleotide and amino acid distances for influenza B virus open reading frame sequences (*n* = 18). Nucleotide and amino acid distances were calculated as described in Figure 1. Axes show amino acid and nucleotide sequence distances; dots correspond to the distances between every two possible sequences in the data set. Color indicates dot density according to the scale.

For each segment, the longest open reading frame was translated into the corresponding protein, and pairwise nucleotide and amino acid distances were calculated for all virus pairs. Heat maps of pairwise distances were generated for each segment (Figure 1 and Figure 2). These plots represented binned two-dimensional density distributions of pairwise comparisons between viral sequences. Each dot corresponded to a pairwise comparison between two viruses, with nucleotide distances plotted on the x-axis and amino acid distances plotted on the y-axis. Color intensity reflected the number of pairwise comparisons falling within each bin, where higher intensity indicated a higher density of observations. Lower density regions correspond to fewer pairwise comparisons occupying the same bin.

For IAV, distinct patterns of divergence were observed among segments. In the PB2, PB1, PA, M1, and NP genes, closely related viruses exhibited predominantly synonymous substitutions, evident as minimal changes at the protein level. In contrast, for more divergent viruses, the relative contribution of nonsynonymous substitutions increased. In other words, amino acid divergence became comparable to nucleotide divergence. This reflected a shift from mainly synonymous changes among closely related viruses to a mixture of synonymous and nonsynonymous substitutions at greater evolutionary distances. One possible technical explanation for this pattern is the accumulation of multiple substitutions at the same nucleotide sites over time (i.e., substitution saturation), which can partially obscure the true number of underlying nucleotide changes. The HA, NA, and NS1 genes exhibited a different pattern. For closely related viruses, the ratios of non-synonymous and synonymous substitutions were similar. However, for more distantly related viruses, elevated non-synonymous divergence likely reflected saturation and heterogeneous selective constraints.

Although H1–H16 and H17–H18 are canonically classified as IAV subtypes, a more rigorous evaluation was undertaken by categorizing them as distinct groups in order to comprehensively assess their underlying genetic divergence. Intragroup pairwise nucleotide and amino acid distances within canonical subtypes (H1–H16) and bat-derived subtypes (H17–H18) did not exceed 25% for PB2, PB1, PA, NP, and M1 genes and 15% for the corresponding proteins. In contrast, intergroup comparisons between H1–H16 and H17–H18 showed substantially higher divergence. Nucleotide differences were at least 30% for M1 and PB1, 35% for NP, and 40% for PB2 and PA; amino acid differences were at least 15% for M1, 20% for PB1, 25% for NP, 30% for PA, and 35% for PB2 (Figure 1). Divergence was significantly more pronounced for NA and NS1 genes. All intragroup pairs remained below these thresholds. Therefore, all genome segments besides HA showed a clear distinction between the two lineages (yellow circles in Figure 1 indicate intergroup pairs). The HA segment did not differentiate the H1–H16 and H17–H18 groups. This supports the hypothesis that the HA gene of H17–H18 viruses originated from an ancestral avian or mammalian IAV through reassortment, while the remaining segments were inherited from a divergent bat-adapted lineage.

For influenza B viruses, pairwise nucleotide distances did not exceed 12%, and amino acid distances were below 5% (for PB2, PB1, PA, NP, M) and 15% (for HA, NA, NS1), respectively (Figure 2). This demonstrates low genetic diversity and supports the current grouping of IBVs into two closely related lineages, Yamagata-like and Victoria-like, rather than distinct subtypes. Notably, the genetic diversity of IBV was lower than within the H1N1 subtype of IAV (Supplementary Figure S1).

**Figure 3:**
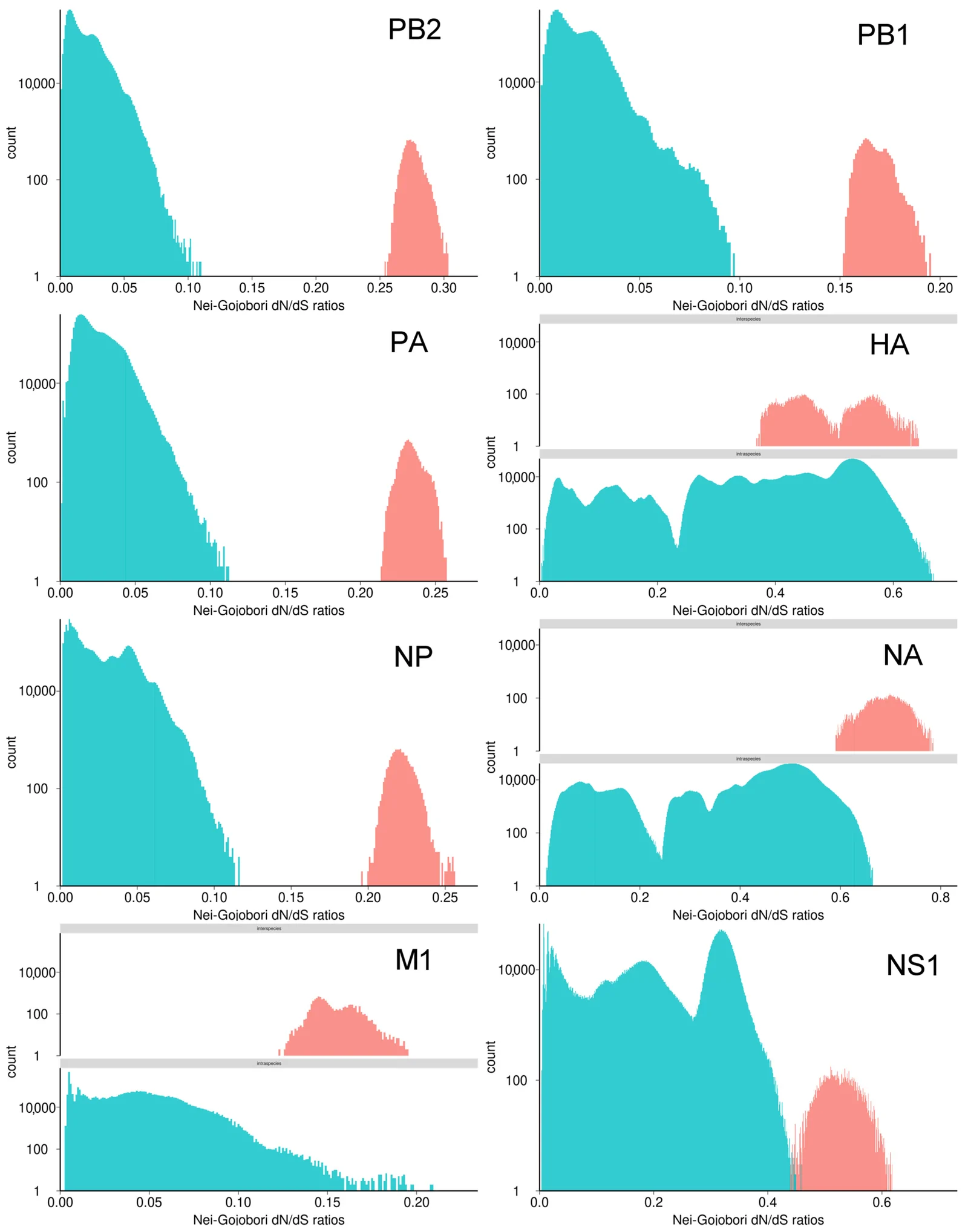
Distribution of pairwise dN/dS ratios among influenza A viruses. Blue—pairs of viruses of the same group (H1–H16 or H17–H18); red—pairs of viruses belonging to different groups (H1–H16 and H17–H18).

To further assess the feasibility of classifying IAV at the subspecies level based on strict criteria, we examined the pairwise dN/dS ratios for all segments and taxa and plotted histograms (Figure 3). In this figure, intragroup values were coloured blue and intergroup values were coloured red. The pairwise dN/dS ratios for the PB2, PB1, PA, and NP segments exhibited a clear bimodal distribution, reflecting intra- and intergroup comparisons. Pairs between canonical IAV subtypes (H1–H16) or between H17 versus H18 exhibited low pairwise dN/dS ratio values (less than 0.12 for the PB2, PB1, PA, and NP genes), consistent with strong purifying selection. In contrast, comparisons between H17–H18 and H1–H16 yielded pairwise dN/dS values of at least 0.25 for PB2, at least 0.15 for PB1, and at least 0.20 for both PA and NP—consistent with a relaxed purifying selection or the effects of divergent functional constraints on a taxonomic level between these groups (Supplementary Table S1). Although the exact quantitative boundaries may be changed with the inclusion of additional isolates, the current dataset demonstrates a consistent separation between canonical and bat IAVs across multiple genomic segments. These values should therefore be interpreted as empirical boundaries derived from the present dataset and specific to *Alphainfluenzavirus* diversification.

These data support the hypothesis that H17–H18 viruses constitute a distinct evolutionary branch within the *Alphainfluenzavirus* genus. Notably, the M1 gene exhibited a clear bimodal distribution of pairwise dN/dS values, comparable to the distributions observed for the PB2, PB1, PA, and NP segments. A small number of intragroup comparisons with pairwise dN/dS values exceeding 0.12 were primarily associated with four strains (A/chicken/Nanjing/908/2009 (H11N2), A/Gramado/LACENRS-1287/2016 (H1N1), A/duck/Eastern_China/40/2007 (H6N2), and A/Helsinki/716/2013 (H3N2)), each contributing numerous high-value pairwise comparisons. To assess the influence of these sequences on the observed M1 pairwise dN/dS distribution, the analysis was repeated after their removal (Supplementary Figure S2). This analysis did not affect the inferred separation pattern, and all thresholds and conclusions presented in this study are based on the complete dataset.

By contrast, the HA, NA, and NS1 genes exhibited much higher dN/dS values that were distributed in a more complicated pattern than in the “conservative” genome segments. These genes experience intense and heterogeneous selection pressures, e.g., immune-driven positive selection or host adaptation, that rendered them more variable within and among groups. High variability likely reflected complex selective pressures and functional constraints, limiting their suitability for threshold-based taxonomy.

Pairwise dN/dS ratios for all segments of influenza B viruses had a unimodal distribution, consistent with their classification within a single species (Supplementary Figure S3). At the same time, segment-specific pattern observed in IAVs was also evident: the HA, NA, and NS1 genes exhibited more heterogeneous pairwise dN/dS distributions than the PB2, PB1, PA, M1, and NP segments, reflecting differences in selective pressures across genome regions.

Analysis of the transition/transversion ratio revealed clear differences between intragroup and intergroup comparisons (Supplementary Figure S4). For sequence pairs with low nucleotide distances, corresponding to intragroup comparisons within H1–H16 or H17–H18, transitions predominated. This is consistant with the accumulation of mostly synonymous substitutions under purifying selection, since transitions are more likely than transversions to preserve amino acid sequence. Conversely, in intergroup comparisons involving H17–H18 and the other subtypes, the proportion of transversions exceeded that of transitions. This shift is consistent with the accumulation of multiple substitutions at nucleotide sites during long-term evolution, which can reduce the apparent contribution of transitions at higher divergence levels. Therefore, the transition/transversion patterns provide additional information on substitution dynamics and are consistent with the deep evolutionary divergence between H17–H18 and H1–H16 viruses.

Maximum-likelihood phylogenetic analyses were performed for each of the eight genomic segments as well as for the concatenated full-genome alignment. In all segment-specific trees except HA, H17–H18 viruses formed a distinct and supported monophyletic clade, clearly separated from canonical H1–H16 influenza A viruses (Supplementary Figures S5–S13). The concatenated genome phylogeny further reinforced this separation, with H17–H18 viruses forming a deeply divergent lineage within the genus *Alphainfluenzavirus* (Supplementary Figures S5–S13).

## 4. Discussion

Representative IAV and IBV genomes were analyzed in order to evaluate quantitative taxonomic criteria. Pairwise genetic distances and dN/dS ratios revealed consistent separation between H1–H16 and H17–H18 for all genome segments with the exception of HA. This pattern suggests that these two groups of viruses separated much longer ago than the viruses within them, were evolving independently, and were not involved in reassortment, with just one exception.

The overlap of these distances between intragroup and intergroup was observed only in the HA segment (Figure 1). The most plausible explanation for this pattern is an ancient reassortment event [14]. Under this scenario, the ancestor of contemporary H17–H18 viruses may have acquired an HA segment related to those present in the H1–H16 group, thereby reducing the apparent genetic distance between the two groups for this particular gene. The evolutionary separation of H17–H18 viruses from canonical H1–H16 influenza A viruses is further supported by maximum-likelihood phylogenetic analyses, which consistently recover H17–H18 as a distinct lineage across genomic segments except HA (Supplementary Figures S5–S13). This topology is consistent with previous phylogenetic observations reported by Tong et al. [14]. With the exception of the HA gene, all other genome segments demonstrated clear segregation of the “canonical” and bat IAV.

The pairwise dN/dS ratio is a derivative of pairwise amino acid and nucleotide distances. However, it may better highlight the difference between taxa by indicating the strength and mode of natural selection acting on protein-coding genes [37]. Conventionally, the pairwise dN/dS ratio is estimated at individual codon sites or across genes using phylogeny-based models applied to multiple sequence alignments. In the present study, however, we employed a pairwise approach in which the numbers of nonsynonymous and synonymous substitutions were calculated for each sequence pair, and their ratio was used as a pairwise dN/dS estimate. This simplified framework was chosen intentionally because the primary objective of the analysis was to compare the overall evolutionary divergence between virus groups. Pairwise estimates allowed direct comparison of intergroup and intragroup substitution patterns without relying on phylogenetic model assumptions and were therefore suited for detecting large-scale evolutionary separation between groups.

For the PB2, PB1, PA, NP, and M genes (in the case of M, except for four viruses described above), the pairwise dN/dS distributions showed a clear bimodal structure separating intergroup and intragroup pairs of IAVs (Figure 3). The pairwise dN/dS values of intergroup comparisons (H1–H16 vs. H17–H18) were consistently greater than intragroup comparisons. This conforms to long-term independent evolution and potentially divergent functional constraints. Interestingly, a comparable threshold has recently been proposed as a quantitative criterion for species demarcation among tick-borne flaviviruses, although it was derived independently for a different viral group [29].

A different pattern was observed for the HA, NA, and NS1 genes. While intergroup virus pairs generally exhibited higher pairwise dN/dS ratios than intragroup pairs, a significant proportion of intergroup comparisons revealed lower pairwise dN/dS values than certain intragroup comparisons (see Figure 3). Pairwise dN/dS values should be interpreted with caution, as they are sensitive to divergence time, substitution saturation, and heterogeneity in selective pressures across genomic segments. Therefore, the dN/dS ratio was not considered as a standalone criterion for species assignment. In the present study, the pairwise dN/dS ratio was used as an additional measure supporting signals of deep evolutionary separation inferred from nucleotide distance and phylogenetic analyses.

A higher dN/dS ratio in HA and NA segments compared to other segments is not surprising, since these proteins are under pressure from the immune system and HA has to adapt to distinct variants of the cellular receptor. Observing a similar profile in the NS1 protein was less expected. The NS1 protein has multiple functions. For instance, NS1 has been demonstrated to regulate viral RNA synthesis, control viral mRNA splicing, enhance viral mRNA translation, and most significantly, suppress host immune responses. Available evidence suggests that the primary function of NS1 is to antagonize host innate antiviral responses. It is important to note that the mechanisms and targets of this response are strain-specific [38]. The dN/dS ratios among IAV suggest that NS1 is under a comparable modifying selection as HA and NA. The second possible explanation for higher non-synonymous variability in NS1 may be related to reassortment. The NA and HA segments are involved in reassortment at a higher rate than others [39]. Concurrently, the NS segment demonstrates the highest reassortment rate among the internal segments [40]. Reassortment has been demonstrated to disrupt genetic linkage and fitness correlations between viral genome segments [41]. Reassortant viruses frequently exhibit reduced fitness until compensatory mutations recover the functional interactions between proteins [41]. The broad spectrum of pairwise dN/dS values occured for HA, NA, and NS may therefore be indicative of adaptive changes that were responsible for the integration of reassorted segments into new genomic environments. Conversely, genomic segments that are involved in reassortment less frequently tend to exhibit stronger functional coadaptation with other proteins, and nonsynonymous substitutions in these genes may be more strongly constrained by purifying selection.

A comparison with IBVs provided further information for interpreting the observed genetic distances in IAVs. IBVs exhibited a significantly lower level of diversity compared to IAVs (Figure 1 and Figure 2). The distances detected between IBVs were comparable to those observed within a single IAV subtype (e.g., H1N1) (Supplementary Figure S1). Thus, IBV is far less variable than even the canonical IAV. Within the limited set of representative IBV genomes analyzed here (*n* = 18), genetic distances were consistently lower than those occured in canonical IAVs, although this comparison is constrained by the substantially smaller and less comprehensive IBV dataset and should not be interpreted as a complete estimate of global IBV diversity. Conversely, the heterogeneity of IAV may potentially result in its subdivision into multiple species if objective quantitative criteria are applied.

Current influenza virus taxonomy largely relies on antigenic properties of the HA and NA proteins. However, the present analysis demonstrates that the genome-wide genetic distances between the H1–H16 and H17–H18 groups are, with few exceptions, substantially greater than intragroup distances. These findings indicate that classification based solely on HA antigenic subtype may not fully reflect deeper evolutionary relationships among influenza viruses. At the same time, the data presented in the current study suggest that bat IAVs H17N10 and H18N11 represent a distinct evolutionary lineage. This pattern is compatible with their consideration as a separate species within the genus *Alphainfluenzavirus*, rather than as divergent subtypes of a single species.

ICTV has historically considered influenza virus species as clusters of strains that replicate as continuous lineages and are capable of genetic reassortment with one another [6]. Extensive intra- and intersubtype reassortment has been documented among canonical H1–H16 influenza A viruses [21,42,43,44]. In contrast, no evidence currently supports ongoing or recent reassortment between the H1–H16 and H17–H18 groups, although a putative ancient reassortment event involving the HA segment has been proposed. Moreover, experimental studies have demonstrated that viable reassortment between these groups is prevented by functional incompatibilities between viral proteins [16,45]. These observations indicate long-term evolutionary independence of the two viral lineages.

The ICTV currently defines virus species as monophyletic groups of viruses that can be distinguished from other species by multiple criteria, including genetic, ecological, and biological characteristics [46]. In other words, modern ICTV taxonomy follows a framework in which no single property is sufficient for species assignment. Accordingly, genome-wide divergence, phylogenetic structure, ecological characteristics, and biological properties (e.g., compatibility of genome segment exchange in segmented viruses) should be viewed as complementary lines of evidence for evolutionary independence. Thus, in the present study, reassortment incompatibility is interpreted as one component of the broader evidence consistent with long-term evolutionary separation between the lineages, together with genome-wide divergence, phylogenetic analyses, and biological differences between canonical and bat influenza A viruses.

The HA segment showed a distinct pattern compared to the remaining genomic segments, as intergroup and intragroup genetic distances overlap. This suggests that HA may have experienced an evolutionary history that is not congruent with the other genes. One plausible explanation is an ancient reassortment event, in which the ancestral HA segment was exchanged between divergent lineages. This hypothesis is also supported by phylogenetic analysis of all segments (Supplementary Figures S5–S13), which is consistent with previous phylogenetic observations reported by Tong et al. [14]. However, alternative scenarios are also possible. In particular, segment-specific selective constraints associated with host receptor interactions may produce similar patterns of phylogenetic incongruence. Therefore, the HA segment is interpreted here as a locus with a distinct evolutionary signal relative to the remaining genome, reflecting either historical reassortment, or long-term evolutionary rate heterogeneity, or a combination of both processes.

In addition to genetic divergence, bat IAVs demonstrate several unusual biological properties. Unlike canonical influenza A viruses, the H17 and H18 hemagglutinins do not bind sialic acid receptors [47]. Viral entry is mediated through interaction with the MHC class II HLA-DR receptor [48]. Furthermore, the N10 and N11 proteins lack sialidase activity and are considered neuraminidase-like glycoproteins with divergent functional properties [49]. Together with the inability of contemporary bat H17 and H18 influenza viruses to reassort with canonical influenza A viruses, these features further emphasize the distinct biological characteristics of this group.

Other influenza A viruses reported in bat hosts (e.g., H9N2 [50]) do not cluster with H17–H18 viruses. Instead, they belong to canonical IAV subtypes with occasional host spillover or adaptation. Consequently, such viruses remain classified within the canonical influenza A virus species and do not affect the delineation of the deeply divergent H17–H18 lineage proposed here.

**Taxonomic proposal.** The results of this study indicate that the H17–H18 viruses represent a deeply divergent group within the genus *Alphainfluenzavirus*. The quantitative criteria identified here (>25% nucleotide divergence across most genomic segments and a consistent separation of pairwise dN/dS distributions with a lower boundary around 0.12) provide an additional line of evidence for evaluating evolutionary divergence within the genus *Alphainfluenzavirus*. Across all eight genomic segments, nucleotide divergence consistently separated H17–H18 viruses from canonical influenza A viruses, with intergroup distances exceeding ~25% in most segments. For PB2, PB1, PA, and NP segments, pairwise dN/dS analyses showed a similar pattern, with intergroup comparisons above a lower boundary of ~0.12, while intragroup values remained below this level. The upper range of intergroup values varied between these gene segments. Taken together, these results support a reproducible multi-segment separation pattern observed in the present dataset that may contribute to reassessing species boundaries within the *Alphainfluenzavirus* genus. In combination with the impossibility of reassortment (i.e., lack of reproductive compatibility) and the unique biological properties of bat influenza viruses, these findings support reconsideration of their taxonomic status. Accordingly, the empirical evidence presented here is consistent with considering H17N10 and H18N11 viruses as representatives of a distinct species, tentatively designated *Alphainfluenzavirus chiropterorum*. Under a revised taxonomic framework, H17N10 and H18N11 designations could be retained as virus names reflecting surface glycoprotein identities, but would no longer imply classification within influenza A virus species. Final nomenclatural decisions, however, remain subject to ICTV approval and implementation of revised taxonomic status.

## Data Availability

Data are available at https://github.com/MarselBrukman/Influenza_alignments (accessed on 26 July 2026).

## Supplementary Materials

The following supporting information can be downloaded at https://www.mdpi.com/article/10.3390/v18080838/s1: Figure S1. Heat map of pairwise nucleotide and amino acid distances for H1N1 influenza A virus open reading frame sequences (*n* = 246). Nucleotide distances were estimated using the Jukes–Cantor model. Amino acid distances were estimated as average substitutions per position using BLOSUM62. Axes show uncorrected amino acid and nucleotide sequence distances; dots correspond to the distances between every two possible sequences in the data set. Color indicates dot density according to the scale. Table S1. Summary of observed nucleotide and amino acid divergence patterns supporting the separation of H17–H18 and H1–H16 influenza A viruses. Figure S2. The distribution of pairwise dN/dS ratios is shown for the M segment of influenza A viruses, except A/chicken/Nanjing/908/2009 (H11N2), A/Gramado/LACENRS-1287/2016 (H1N1), A/duck/Eastern_China/40/2007 (H6N2), and A/Helsinki/716/2013 (H3N2). Pairs of viruses belonging to the same group (H1–H16 or H17–H18) are represented in blue, while pairs belonging to different groups (H1–H16 and H17–H18) are represented in red. Figure S3. Distribution of pairwise dN/dS ratios among influenza B viruses. Figure S4. Pairwise nucleotide distances and the proportions of transitions (red) and transversions (blue) among IAV subtypes. The x-axis shows the p-distances (pairwise nucleotide distances) between virus sequences. The y-axis shows the proportion of transitions and transversions for each pair of sequences, totaling 100%. Each genome pair is represented by two dots, red dots correspond to transitions and blue dots to transversions. The group of dots forming right clusters (circled) reflects comparisons between bat subtypes H17–H18 and other IAV subtypes. For intragroup pairs, transitions predominate, consistent with the accumulation of predominantly synonymous substitutions. In intergroup pairs, the proportion of transversions increases and approaches or exceeds that of transitions, indicating a higher level of genetic divergence between bat and canonical subtypes. Figure S5. Midpoint-rooted maximum-likelihood phylogenetic tree (PB2) indicating the position of H17/H18 viruses. Their grouping was supported by high bootstrap values. The remaining influenza A virus sequences also showed well-supported clustering. Figure S6. Midpoint-rooted maximum-likelihood phylogenetic tree (PB1) indicating the position of H17/H18 viruses. Their grouping was supported by high bootstrap values. The remaining influenza A virus sequences also showed well-supported clustering. Figure S7. Midpoint-rooted maximum-likelihood phylogenetic tree (PA) indicating the position of H17/H18 viruses. Their grouping was supported by high bootstrap values. The remaining influenza A virus sequences also showed well-supported clustering. Figure S8. Midpoint-rooted maximum-likelihood phylogenetic tree (HA) indicating the position of H17/H18 viruses. In contrast to other segments, this tree did not show a separation of H17/H18 viruses from the canonical IAV subtypes. Figure S9. Midpoint-rooted maximum-likelihood phylogenetic tree (NP) indicating the position of H17/H18 viruses. Their grouping was supported by high bootstrap values. The remaining influenza A virus sequences also showed well-supported clustering. Figure S10. Midpoint-rooted maximum-likelihood phylogenetic tree (NA) indicating the position of H17/H18 viruses. Their grouping was supported by high bootstrap values. The remaining influenza A virus sequences also showed well-supported clustering. Figure S11. Midpoint-rooted maximum-likelihood phylogenetic tree (M1) indicating the position of H17/H18 viruses. Their grouping was supported by high bootstrap values. The remaining influenza A virus sequences also showed well-supported clustering. Figure S12. Midpoint-rooted maximum-likelihood phylogenetic tree (NS1) indicating the position of H17/H18 viruses. Their grouping was supported by high bootstrap values. The remaining influenza A virus sequences also showed well-supported clustering. Figure S13. Midpoint-rooted maximum-likelihood phylogenetic tree (concatenated genome alignment) indicating the position of H17/H18 viruses. Their grouping was supported by high bootstrap values. The remaining influenza A virus sequences also showed well-supported clustering.
