# Supplementary material for: Assessment of Quantitative Genetic Distances Supports the Separation of H17N10 and H18N11 Subtypes of Influenza A Virus into a Distinct Species
Source: Viruses. 2026 Jul 30;18(8):838. doi: 10.3390/v18080838 (PMC13517615; doi:10.3390/v18080838)
Supplement: Supplementary file 1 [file viruses-18-00838-s001.zip › Supplementary Figure captions.pdf]

Supplementary Figure S1. Heat map of pairwise nucleotide and amino acid distances for H1N1 influenza A virus open reading frame sequences (n = 246). Nucleotide distances were estimated using the Jukes-Cantor model. Amino acid distances were estimated as average substitutions per position using BLOSUM62. Axes show uncorrected amino acid and nucleotide sequence distances; dots correspond to the distances between every two possible sequences in the data set. Color indicates dot density according to the scale.

Supplementary Figure S2. The distribution of pairwise dN/dS ratios is shown for the M segment of influenza A viruses, except A/chicken/Nanjing/908/2009 (H11N2), A/Gramado/LACENRS-1287/2016 (H1N1), A/duck/Eastern_China/40/2007 (H6N2), and A/Helsinki/716/2013 (H3N2). Pairs of viruses belonging to the same group (H1–H16 or H17–H18) are represented in blue, while pairs belonging to different groups (H1–H16 and H17–H18) are represented in red.

Supplementary Figure S3. Distribution of pairwise dN/dS ratios among influenza B viruses.

Supplementary Figure S4. Pairwise nucleotide distances and the proportions of transitions (red) and transversions (blue) among IAV subtypes. The x-axis shows the p-distances (pairwise nucleotide distances) between virus sequences. The y-axis shows the proportion of transitions and transversions for each pair of sequences, totaling 100%. Each genome pair is represented by two dots, red dots correspond to transitions and blue dots to transversions. The group of dots forming right clusters (circled) reflects comparisons between bat subtypes H17–H18 and other IAV subtypes. For intragroup pairs, transitions predominate, consistent with the accumulation of predominantly synonymous substitutions. In intergroup pairs, the proportion of transversions increases and approaches or exceeds that of transitions, indicating a higher level of genetic divergence between bat and canonical subtypes.

Supplementary Figure S5. Midpoint-rooted maximum-likelihood phylogenetic tree (PB2) indicating the position of H17/H18 viruses. Their grouping was supported by high bootstrap values. The remaining influenza A virus sequences also showed well-supported clustering.

Supplementary Figure S6. Midpoint-rooted maximum-likelihood phylogenetic tree (PB1) indicating the position of H17/H18 viruses. Their grouping was supported by high bootstrap values. The remaining influenza A virus sequences also showed well-supported clustering.

Supplementary Figure S7. Midpoint-rooted maximum-likelihood phylogenetic tree (PA) indicating the position of H17/H18 viruses. Their grouping was supported by high bootstrap values. The remaining influenza A virus sequences also showed well-supported clustering.

Supplementary Figure S8. Midpoint-rooted maximum-likelihood phylogenetic tree (HA) indicating the position of H17/H18 viruses. In contrast to other segments, this tree did not show a separation of H17/H18 viruses from the canonical IAV subtypes.

Supplementary Figure S9. Midpoint-rooted maximum-likelihood phylogenetic tree (NP) indicating the position of H17/H18 viruses. Their grouping was supported by high bootstrap values. The remaining influenza A virus sequences also showed well-supported clustering.

Supplementary Figure S10. Midpoint-rooted maximum-likelihood phylogenetic tree (NA) indicating the position of H17/H18 viruses. Their grouping was supported by high bootstrap values. The remaining influenza A virus sequences also showed well-supported clustering.

Supplementary Figure S11. Midpoint-rooted maximum-likelihood phylogenetic tree (M1) indicating the position of H17/H18 viruses. Their grouping was supported by high bootstrap values. The remaining influenza A virus sequences also showed well-supported clustering.

Supplementary Figure S12. Midpoint-rooted maximum-likelihood phylogenetic tree (NS1) indicating the position of H17/H18 viruses. Their grouping was supported by high bootstrap values. The remaining influenza A virus sequences also showed well-supported clustering.

Supplementary Figure S13. Midpoint-rooted maximum-likelihood phylogenetic tree (concatenated genome alignment) indicating the position of H17/H18 viruses. Their grouping was supported by high bootstrap values. The remaining influenza A virus sequences also showed well-supported clustering.
