# Supplementary material for: Assessment of Quantitative Genetic Distances Supports the Separation of H17N10 and H18N11 Subtypes of Influenza A Virus into a Distinct Species
Source: Viruses. 2026 Jul 30;18(8):838. doi: 10.3390/v18080838 (PMC13517615; doi:10.3390/v18080838)
Supplement: Supplementary file 1 [file viruses-18-00838-s001.zip › Supplementary Table S1.pdf]

Supplementary Table S1. Summary of observed nucleotide and amino acid divergence patterns supporting the separation of H17–H18 and H1–H16 influenza A viruses.

Gene	Upper boundary of intragroup nucleotide divergence (%)	Lower boundary of intergroup nucleotide divergence (%)	Upper boundary of intragroup pairwise dN/dS	Lower boundary of intergroup pairwise dN/dS
PB2	<25	>40	<0.12	>0.25
PB1	<25	>30	<0.10	>0.15
PA	<25	>40	<0.12	>0.20
HA	n/a	n/a	<0.70	>0.35
NP	<25	>35	<0.12	>0.17
NA	n/a	n/a	<0.70	>0.55
M1	<20	>30	<0.22	>0.12
NS1	n/a	n/a	<0.45	>0.40
